# Endoscopic Ultrasound-Guided Laser Ablation Using a Diffusing Applicator for Locally Advanced Pancreatic Cancer Treatment

**DOI:** 10.3390/cancers14092274

**Published:** 2022-05-02

**Authors:** Seonghee Lim, Van Gia Truong, Jongman Choi, Hye Jung Jeong, Sun-Ju Oh, Jin-Seok Park, Hyun Wook Kang

**Affiliations:** 1 Industry 4.0 Convergence Bionics Engineering, Pukyong National University, Busan 48513, Korea; seonghee@pukyong.ac.kr (S.L.); truonggia@pukyong.ac.kr (V.G.T.); cjman930@pukyong.ac.kr (J.C.); 2 Digestive Disease Center, Department of Internal Medicine, Inha University School of Medicine, Incheon 22332, Korea; jhj4358@naver.com; 3 Department of Pathology, Kosin University College of Medicine, Busan 49267, Korea; sjoh@kosin.ac.kr; 4 Marine—Integrated Biomedical Technology Center and Department of Biomedical Engineering, Pukyong National University, Busan 48513, Korea

**Keywords:** laser ablation, diffusing applicator, endoscopy ultrasound, energy level, pancreatic cancer

## Abstract

**Simple Summary:**

Pancreatic cancer (PC) is one of the most lethal cancers; caused by family history, obesity, diabetes, and smoking, it has a 2–9% five-year survival rate. However, patients diagnosed by endoscopic ultrasound (EUS) already have an advanced stage of PC, indicating the difficulty of surgical resection. Recently, laser ablative treatment with a diffusing applicator has been proven to be feasible for treating advanced PC. Despite the potential application for treating PC, further evaluation of acute and chronic tissue responses are essential to determine the efficacy and safety of diffusing applicator under EUS guidance. In this study, EUS-guided diffusing applicator-assisted laser ablation was evaluated to quantify the extent of the tissue response after the delivery of various energy levels.

**Abstract:**

Endoscopic ultrasound (EUS)-guided cylindrical interstitial laser ablation (CILA) procedures can be used to treat unresectable pancreatic cancer (PC). The aim of this study was to investigate the acute responses of pancreatic tissue after EUS-guided CILA in vivo in porcine models. Eight pigs were tested to compare the effects of different energy levels on pancreatic tissue ablation. A 1064 nm laser system was used to deliver 5 W through a diffusing applicator. The EUS-guided CILA was performed under four different energies: 200, 400, 600, and 800 J. Three days after the experiments, histological analysis was performed. The CILA consistently generated circular coagulated necrosis (CN) in the cross-sectioned pancreatic tissue. The ablation diameter was linearly dependent on the total energy delivery. The area of the CN initially increased with total energy delivery but became saturated at 600 J. The width of the degenerative parenchyma (DP) in the native tissue beyond the CN region increased with the total energy up to 600 J, and then decreased afterward. EUS-guided CILA can be a feasible approach for treating PC. Further animal studies will investigate the chronic responses of the pancreatic tissue to examine the efficacy and safety of the proposed method for clinical translation.

## 1. Introduction

Pancreatic ductal adenocarcinoma (PDAC), the most common type of pancreatic cancer (PC), is a highly fatal disease. It is generally caused by family history, obesity, diabetes, and/or smoking, and it has a 2–9% five-year survival rate [1]. Although PC can normally be diagnosed using endoscopic ultrasound (EUS) or endoscopic retrograde cholangiography, unresectable PC (URPC) or locally advanced PC (LAPC) are associated with difficulties in completing treatment and poor prognoses [2,3]. Most patients already have URPC and LAPC when they are diagnosed with late symptoms, leading to difficulty in approaching surgical resection [3,4]. Although chemotherapy is usually utilized for both URPC and LAPC patients, patients often suffer because of the low success rate (up to 25%) and severe side effects [5]. Focal ablation is an alternative therapeutic option for treating both URPC and LAPC patients [4]. Recently, ablative therapies, such as radiofrequency ablation (RFA), irreversible electroporation, and interstitial laser ablation (ILA), have been widely studied for treating URPC and LAPC [6,7,8].

Previous studies have reported the development of EUS-guided cylindrical ILA (CILA), which uses a diffusing applicator to ablate pancreatic tissue in a predictable manner [9,10]. Unlike a flat fiber, the diffusing applicator can help create a circular shape of tissue ablation with minimal thermal injury to the adjacent tissue [9,10]. Despite the potential efficacy of EUS-guided CILA, studies have generally focused on evaluating the immediate response of the pancreatic tissue after laser irradiation. Thus, further evaluation of the acute and chronic tissue responses to laser treatment is necessary to determine the efficacy and safety of EUS-guided CILA for clinical translation. 

The current study aimed to investigate the acute responses of pancreatic tissue after laser treatment and to comparatively quantify the extent of tissue ablation after the delivery of various energy levels. We hypothesized that the EUS-guided CILA could control the dimensions of the treated pancreatic tissue by adjusting the total energy delivery. Four energy levels were applied to identify the optimal conditions for EUS-guided CILA in vivo in porcine models. Histological analysis was performed to evaluate the acute responses of the pancreatic tissue after CILA.

## 2. Materials and Methods

### 2.1. Devices and Laser System

**Figure 1:**
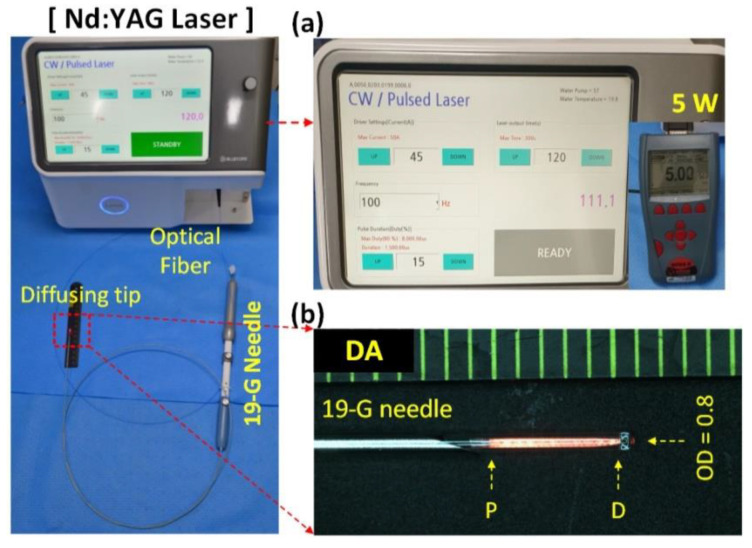
Endoscopic ultrasound (EUS)-guided laser ablation (LA) on pancreatic tissue in vivo in porcine models with diffusing applicator (DA) through 19 G biopsy needle: (**a**) schematic of customized Nd:YAG laser system and (**b**) uniform HeNe light distribution along the DA (P = proximal and D = distal ends; OD = outer diameter of DA).

A diffusing applicator (core diameter = 400 μm; TeCure, Inc., Busan, Korea) was prepared by micromachining a fiber tip surface. An active element of 5 mm was fabricated to irradiate the laser light circumferentially for tissue ablation (Figure 1). A 19-G biopsy needle (Cook Medical Inc., Bloomington, IN, USA) was used to puncture the porcine pancreatic tissue through an endoscope channel, and the applicator was inserted into the needle. An endoscope with an EU-ME2 ultrasound system (GF-UCT260, Olympus Medical Co., Ltd., Tokyo, Japan) was employed to confirm the positions of both the biopsy needle and the diffusing applicator. The diffusing applicator was coupled with a customized 1064 nm laser system (Bluecore Company, Busan, Korea) to deliver a laser power of 5 W to the porcine pancreatic tissue for 40, 80, 120, and 160 s (Figure 1). Thus, four different energy groups (200, 400, 600, and 800 J) were evaluated and compared.

### 2.2. In Vivo Porcine Tests

A total of eight male pigs (Sus scrofa domesticus; 30–35 kg) were used to conduct the in vivo EUS-guided CILA tests (N = 2 per energy group). All animal experimental procedures followed the Korean National Institutes of Health guidelines and were approved by the Institutional Animal Care and Use Committee of Knotus, Korea (permit number: IACUC 21-KE-442). Prior to the ablation tests, all the animals were injected with a 0.1 mL/kg mixture of zoletil and xylazine (1:1 ratio) for general anesthesia. Subsequently, a ventilator provided isoflurane (1–2%) and oxygen (2 L/min) for the animals to maintain general anesthesia during the experiments. A 19 G biopsy needle was initially inserted through the endoscopic channel, and the stomach was punctured under endoscopic visualization to reach the pancreatic tissue. After ultrasound imaging confirmed the location of the needle, the needle was moved back 10 mm, and the diffusing applicator was inserted through the inner lumen of the biopsy needle. Video S1 provides an overview of the procedure of inserting the diffusing applicator into the 19 G biopsy needle. To compare the various energy conditions (200, 400, 600, and 800 J), 1064 nm laser light was delivered to each tissue through the diffusing applicator. Three days after the experiments, all the animals were euthanized by bloodletting in axillary artery and vein under anesthesia using an intramuscular injection of a 0.1 mL/kg zoletil–xylazine mixture (ratio 1:1) for post-operative analysis. Blood tests were conducted three times during animal monitoring.

### 2.3. Histological Analysis

All the treated samples of the pancreas were extracted three days after the experiments and were fixed in 10% formalin for a week. Then, the tissue samples were sectioned into 5 μm slices and stained with hematoxylin and eosin. The stained tissue slides were scanned using a digital slide scanner (Motic Easy Scan Pro Digital Slide Scanner, Motic Asia Corp., Kowloon, Hong Kong). The Motic “DSAssistant” software was used to observe and photograph the biological and structural changes in the treated tissue. The tissue slides were selected from three ablation points in the pancreatic tissue for each group. Then, the extent of laser ablation (LA) was estimated quantitatively by measuring the ablation diameter and necrotic area in the treated pancreatic tissue. The distance between the inflammatory band located at the edge of the central necrotic area and native tissue (NT) was measured to assess the width of the degenerative parenchyma (DP), as this indicates the ablative impact on the NT beyond the necrotic area.

### 2.4. Statistical Analysis

All the data and graphs are presented as means ± standard deviation for quantitative evaluation. For the nonparametric tests, SPSS software (IBM SPSS Statistics 22, IBM, Armonk, NY, USA) was used to perform Kruskal–Wallis and Mann–Whitney U tests to compare multiple groups and two groups, respectively. *p* < 0.05 represents statistical significance.

## 3. Results

Blood tests were conducted pre- and post-operatively and before euthanasia for animal monitoring (Table 1). Although relatively higher concentrations of digestive enzymes, such as amylase and lipase, were observed before euthanasia relative to the pre-operation stage, all the measured values remained within the normal range.

**Figure 2:**
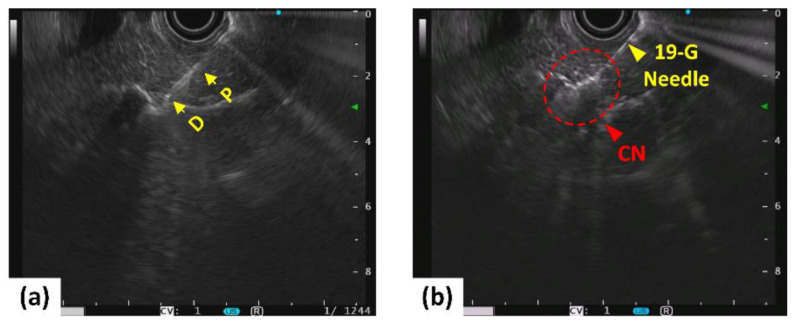
EUS-guided LA on pancreatic tissue in porcine models before (left; (**a**)) and after (right; (**b**)) irradiation at 5 W for 160 s (P = proximal and D = distal ends of diffusing applicator; CN: coagulative necrosis).

Figure 2 shows the EUS images captured after insertion of both the biopsy needle and diffusing applicator into porcine pancreatic tissue. Prior to laser irradiation, the position of the diffusing applicator in the pancreatic tissue was confirmed by EUS visualization (Figure 2a). Figure 2b presents whitish regions around the diffusing applicator in the treated tissue, representing coagulated necrosis (CN) after 5 W irradiation for 160 s. Video S2 shows the EUS monitoring of the CILA in the pancreas in a porcine model.

**Figure 3:**
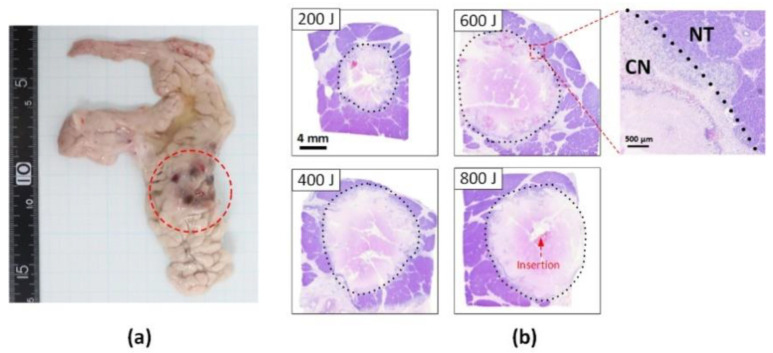
Assessments of thermal ablation in in vivo pancreatic tissue after 5 W LA at various energy levels: (**a**) gross pathology image after 400 J delivery (red circle = ablated area) and (**b**) transverse histological images acquired from tissue treated at various energy levels (CN = coagulation necrosis and NT = native tissue).

Figure 3 shows a photograph of the gross pancreatic tissue (Figure 3a) and histological images (Figure 3b) after 5 W irradiation (400 J delivery). The red dotted circle in Figure 3a shows the discoloration (tanned color) of the thermally treated tissue. Figure 3b shows the cross-sectional histology images acquired for the various energy levels. The CN can be more vividly observed as discoloration in the histology images by demarcating the boundary between the NT and CN (magnified image in the top right corner). Regardless of the energy level, the laser treatment created a circular shape of CN around the diffusing applicator in the treated pancreatic tissue, representing a circumferential thermal ablation. The extent of CN increased with the amount of energy delivered.

**Figure 4:**
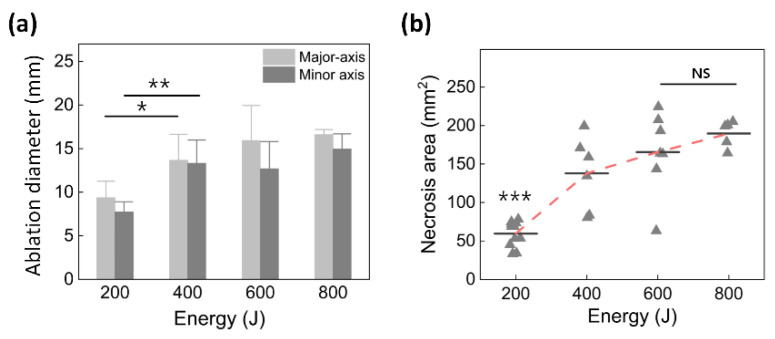
Quantitative comparison of (**a**) ablation diameter (KW *p* < 0.001; * MU *p* < 0.05 and ** MU *p* < 0.01 vs. 400 J) and (**b**) necrosis area measured from histology images of treated pancreatic tissue as function of total energy delivery (KW *p* < 0.001; *** MU *p* < 0.001 vs. 400 J; NS: no significant difference between 600 J and 800 J, *p* = 0.43).

Figure 4 shows a quantitative comparison of the ablation diameter (Figure 4a) and necrosis area (Figure 4b) as measured from the treated pancreatic tissues. Figure 4a shows the ablation diameter in terms of the major axis and minor axis of the ablated region. The ablation diameters in the major and minor axes increased with the applied energy. Figure 4b presents the necrotic area of the pancreatic tissue as a function of the total energy. The necrosis area initially increased with the total energy delivery but became saturated at 600 J (165.6 ± 53.1 mm^2^ for 600 J vs. 169.2 ± 52.5 mm^2^ for 800 J; *p* = 0.43).

**Figure 5:**
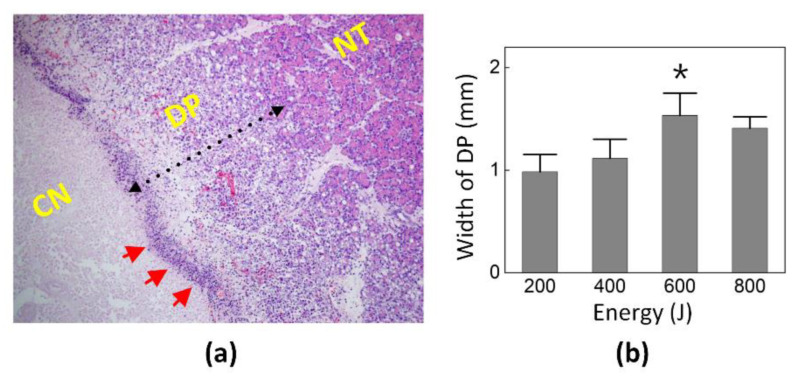
Histological assessments of parenchymatous degeneration in pancreatic tissue after EUS-guide LA: (**a**) Hematoxylin and eosin (HE)-stained histology image (100×) showing boundary between native tissue (NT) and coagulation necrosis (CN) and (**b**) quantitative comparison of width of degenerative parenchyma (DP) in four energy groups. Red arrows indicate an inflammatory band (* MU *p* < 0.05 vs. 200 J and 400 J).

Figure 5a illustrates histological assessments of the parenchymal degeneration in the pancreatic tissue after the EUS-guided CILA. The inflammatory cells and debris formed an inflammatory band along the boundary of the CN. The LA affected the NT beyond the CN region by causing degeneration of pancreatic acinar cells between the inflammatory band and NT. The width of the DP was measured on the basis of the remaining normal pancreatic parenchyma to the inflammatory band at the edge of CN to assess the degenerative change. Figure 5b compares the width of the DPs in the various energy groups. The width of the DP increased with the total energy up to 600 J and then decreased at 800 J. All the energy groups had less than 2 mm of DP, and the 600 J group created the largest DP width (1.5 ± 0.2 mm; *p* < 0.05 vs. 200 and 400 J).

## 4. Discussion

EUS-guided ablation therapies have been widely used as alternative treatments for PC, mainly owing to their advantages regarding real-time imaging and focal ablation. Multiple case reports on EUS-guided RFA have demonstrated results from clinical trials using both the Habid^TM^ EUS-RFA probe (EMcision, London, UK) and an EUS-RFA needle (Taewoong Medical Co., Ltd., Gyeonggi-do, South Korea) [11,12]. EUS-RFA is usually performed on patients for whom chemotherapy is unsuitable or after ineffective chemotherapy owing to a large-sized tumor (i.e., 2–5 cm^3^). Crio et al. reported EUS-guided RFA in eight patients with locally advanced PDAC, and the mean volume of thermal necrosis only accounted for 30% of the tumor mass (range 5.8–73.5%) after one-day and one-month treatments [13]. Owing to the small ablation zone (i.e., 0.13–0.34 cm^3^), multiple punctures with the RFA needles were required to cover a large tumor, eventually increasing undesired complications, such as mild abdominal pain and peripancreatic effusion [11,14]. In other studies, an alternative approach, i.e., EUS-guided LA using a thin flexible optical fiber (i.e., 300-um fiber, Echolaser, Florence, Italy), has been performed to ablate large-sized tumors (up to 2.3 cm^3^ in volume with a single application) [15,16]. After ex vivo and in vivo studies, the first clinical trial from Italy was performed on nine patients with stage IIb–III PDAC [7]. At post-EUS-LA, the mean ablation lesion was 35.4 mm (range 21–45 mm) at low-power settings (2, 3, and 4 W at a 1064 nm wavelength). The mean overall survival was only 7.4 months (range 29–662 days). Another study (NCT03784417) was performed on 20 patients to evaluate the safety and effectiveness of EUS-LA for complete tumor ablation [12]. Both Jiang et al. and Matteo et al. reported that EUS-guided LA may be a potential alternative method and is well-tolerated in patients for whom chemotherapy is ineffective or unsuitable [7,17].

Instead of a flat fiber, the current study used a diffusing applicator with cylindrical light emission for circumferential LA of the pancreatic tissue, aiming to generate uniform ablation lesions (i.e., in a predictable manner) and a large volume of thermal capability (i.e., large tissue coverage) for in vivo, ex vivo, and numerical simulations [9,10]. During the current experiments, no technical difficulties were found when the diffusing applicator was inserted through the 19 G biopsy needle on eight mini-pigs (Figure 2 and Video S1). All the animals survived for three days after the treatment without complications, such as poor feeding and/or acute bleeding, as confirmed by the fact that the parameters of the blood tests remained within normal ranges (Table 1). In addition, the in vivo study confirmed that the thermal damage expanded radially around the diffusing applicator in the treated pancreatic tissue and increased linearly with the total energy delivery (Figure 3 vs. Figure 4). Therefore, EUS-guided CILA with a large uniform ablation lesion shows the ability to create a large uniform ablation lesion and thus treat pancreatic tissue in a predictable manner.

EUS guidance is a beneficial technique for defining the position of both the biopsy needle and the diffusing tip in the pancreatic tissue so as to prevent adverse injury to the surrounding organs during laser irradiation. However, in this study, the position of the diffusing applicator was sometimes difficult to visualize during the EUS monitoring owing to the low reflection of the ultrasound signals. Therefore, to ensure the accurate positioning of the diffusing applicator, a small highly reflective material should be attached to the proximal end of the applicator tip to induce the reflected US signals for EUS detection during the procedure. Additionally, an intravenous SonoVue^TM^ injection followed by a saline solution flush should be performed before and after LA to enhance the contract-EUS image for optimal treatment performance in terms of the ablation lesion and position of the diffusing applicator [18,19].

The histological analysis demonstrated a well-defined demarcation between the native and necrotic tissue three days after the EUS-guided CILA (Figure 3 vs. Figure 5). The current findings confirmed that the pancreatic tissue ablated with a less than 1.5 mm margin of the DP (maximum DP width = 1.5 ± 0.2 mm at 600 J energy; Figure 5) after the three-day treatment. This confirms the treatment safety for normal tissue beyond the tumor in a clinical situation, according to the study of Hank et al. [20]. In our previous study [10], the preservation of the surrounding tissue, such as the biliary duct and blood vessels, was found through histological analysis, i.e., by comparing the abundance of elastic fibers in their structures after CILA relative to soft tissue. Therefore, CILA can guarantee minimal or no thermal injury to the surrounding tissues during and after PC treatment, including blood vessels (i.e., the superior mesenteric artery and portal vein) and/or peripheral tissue structures.

In addition, the current study demonstrates the acute responses of the pancreatic tissue 3 days after EUS-guided CILA. However, further studies should be performed to confirm the long-term safety and efficacy of EUS-guided CILA for clinical translations. Thus, various assessments will be performed in 2-month survival studies to compare acute and chronic responses in terms of the extent of ablation and changes in digestive enzymes (i.e., amylase and lipase) [21,22]. Furthermore, porcine models with pancreatic tumors will be developed to reflect the tissue consistency and perfusion effect in the cancer tissue and evaluate both necrotic and apoptotic effects of EUS-guided CILA using immunohistochemistry for clinical practice.

Although the current study showed the clinical feasibility of EUS-guided CILA for treating pancreatic tissue, limitations remain. For example, the number of mini pigs was relatively small (N = 8) compared with that of other investigations [23,24]. The laser power was low, i.e., only applied at 5 W at various energy levels, to prevent potential adjacent damage to the surrounding tissues. Thus, further studies on a large number of pigs are necessary to identify the optimal treatment conditions for CILA. In addition, normal porcine pancreatic tissue hardly reflects the various features of human pancreatic tissue. Hence, for clinical translations, tumor-developed animal models for both soft and hard tumors will be investigated with EUS-guided CILA to evaluate complete tumor removal as well as the acute and chronic responses after CILA.

## 5. Conclusions

The current in vivo studies confirmed that EUS-guided LA using a diffusing applicator generates a uniform, predictable ablation lesion and can regulate ablation areas by adjusting the total energy delivery. EUS-guided CILA can be a feasible way to effectively treat PC. Further long-term in vivo studies will investigate the complete removal of the pancreatic tumor and the healing response of the pancreatic tissue to ensure the enhanced efficacy and safety of the proposed EUS-guided CILA for clinical translation.

## Figures and Tables

**Table 1:** Summary of blood tests at various time points (pre-operation, post-operation, and before euthanasia).

	Mean (Range)		
	Pre-op	Post-op	Before Euthanasia
WBCB (×103 cells/μL)	22.0 (15.1–31.6)	21.1 (13.6–29.6)	22.6 (15.4–32.2)
RBC (×106 cells/μL)	6.1 (5.6–6.7)	5.9 (5.1–6.7)	6.2 (5.5–6.9)
measHGB (g/dL)	9.6 (8.8–11.1)	9.3 (8.5–10.3)	9.8 (8.3–11.5)
PLT (×103 cells/μL)	451.4 (249.0–686.0)	400.1 (155.0–603.0)	421.1 (168.0–595.0)
Creatinine (mg/dL)	0.9 (0.7–1.1)	1.1 (0.8–1.7)	1.2 (0.2–3.0)
T. Bil (mg/dL)	0.6 (0.2–1.0)	0.5 (0.2–0.9)	0.6 (0.2–1.1)
Amylase (U/L)	2234.1 (1539.0–3310.0)	1998.0 (1511.0–2780.0)	3789.4 (1435.0–9410.0)
Lipase (U/L)	7.0 (1.2–11.7)	6.5 (1.6–17.0)	156.8 (10.5–617.4)

## Data Availability

The data presented in this study is available on request from the corresponding author.

## Supplementary Materials

The following supporting information can be downloaded at: https://www.mdpi.com/article/10.3390/cancers14092274/s1, Video S1: Overview of inserting the diffusing applicator into a 19 G biopsy needle, Video S2: Endoscopic ultrasound (EUS) monitoring of cylindrical interstitial laser ablation (CILA) on pancreas in porcine model.
